# HIV Infection, Antiretroviral Drugs, and the Vascular Endothelium

**DOI:** 10.3390/cells13080672

**Published:** 2024-04-12

**Authors:** Georgette D. Kanmogne

**Affiliations:** Department of Pharmacology and Experimental Neuroscience, College of Medicine, University of Nebraska Medical Center, Omaha, NE 68198-5800, USA; gkanmogne@unmc.edu

**Keywords:** HIV comorbidities, antiretrovirals, protease inhibitors, NRTIs, NNRTIs, INSTIs, maraviroc, endothelial activation, vascular injury and function

## Abstract

Endothelial cell activation, injury, and dysfunction underlies the pathophysiology of vascular diseases and infections associated with vascular dysfunction, including human immunodeficiency virus (HIV) and acquired immunodeficiency syndrome. Despite viral suppression with combination antiretroviral therapy (ART), people living with HIV (PLWH) are prone to many comorbidities, including neurological and neuropsychiatric complications, cardiovascular and metabolic diseases, premature aging, and malignancies. HIV and viral proteins can directly contribute to the development of these comorbidities. However, with the continued high prevalence of these comorbidities despite viral suppression, it is likely that ART or some antiretroviral (ARVs) drugs contribute to the development and persistence of comorbid diseases in PLWH. These comorbid diseases often involve vascular activation, injury, and dysfunction. The purpose of this manuscript is to review the current literature on ARVs and the vascular endothelium in PLWH, animal models, and in vitro studies. I also summarize evidence of an association or lack thereof between ARV drugs or drug classes and the protection or injury/dysfunction of the vascular endothelium and vascular diseases.

## 1. Introduction

The vascular endothelium consists of a single layer of endothelial cells (ECs) (Table 1) that lines the lumen of blood vessels; it functions as a semi-permeable barrier between circulating blood and tissues, regulating blood flow and clotting, vascular permeability, and remodeling [1,2]. The endothelium regulates vascular tone by producing and releasing vasoactive mediators, including vasodilators such as nitric oxide, prostacyclin, bradykinin, and endothelium-derived hyperpolarizing factor, and vasoconstrictors such as endothelin-1, angiotensin-II, thromboxane-A2, and reactive oxygen species (ROS) [1,2,3]. In addition to regulating vascular homeostasis, signaling in vascular ECs is often associated with the production of angiocrine factors that regulate antigen recognition and immune surveillance by effector T-cells [4,5], cell proliferation and tissue regeneration [6,7], and metastasis [8]. Endothelial activation, dysfunction, and injury often involve an imbalance in the production of vasodilators and vasoconstrictors, including the suppression/reduced production of vasodilators, increased production of vasoconstrictors, ROS, and inflammatory molecules [2,3,9,10]. Endothelial activation, injury, and dysfunction underlies the pathology of vascular diseases and infections associated with vascular dysfunction, including human immunodeficiency virus (HIV) infection and acquired immunodeficiency syndrome (AIDS) [11,12,13,14,15,16,17].

The advent of combination antiretroviral therapy (ART) and success in HIV/AIDS treatment over the past three decades has dramatically reduced mortality and changed HIV infection into a chronic disease, with HIV-infected patients now having longer life expectancy and being able to live for decades with suppressed viremia. However, despite viral suppression with ART, people living with HIV (PLWH) are prone to many comorbidities, including neurological and neuropsychiatric complications [18,19,20,21], cardiovascular and metabolic diseases [21,22,23,24,25], premature aging [26,27,28], and malignancies [29,30,31]. It has been demonstrated that HIV and viral proteins can be directly involved in the development of these comorbid diseases [32,33,34,35]. With continued high prevalence of these comorbidities despite viral suppression, one important question is whether ART or some antiretroviral (ARVs) drugs contribute to the development and persistence of comorbid diseases in PLWH. These comorbid diseases often involve vascular activation, injury, and dysfunction. The purpose of this manuscript is to review the current literature on ARVs and the vascular endothelium in PLWH, as well as studies using animal models and those performed in vitro. I further synthesize and summarize evidence of an association or lack thereof between ARV drugs or drug classes and the protection or injury/dysfunction of the vascular endothelium and vascular diseases.

## 2. Evidence from Human Studies

### 2.1. ART, Vascular Endothelium Injury and Dysfunction, and Markers of Vascular Diseases in PLWH

Several human studies have shown that ART use in PLWH is associated with the injury and/or dysfunction of the vascular endothelium. A study of HIV+ humans who had been on nucleoside reverse transcriptase inhibitors (NRTIs) for one year or less showed evidence of sub-clinical atherosclerosis (increased intima media thickness of carotid artery [cIMT]) compared to PLWH who had never received NRTIs [36]. A comparative study of HIV+ patients on ART, healthy seronegative controls, and seronegative controls with established coronary artery disease (CAD) showed that HIV+ patients had higher cIMT compared to healthy controls, and HIV+ subjects and seronegative individuals with CAD had similar cIMT values [37]. In another study comparing HIV+ patients with and without CAD (HIV+CAD+ and HIV+CAD-) and demographically matched seronegative controls with and without CAD (HIV-CAD+ and HIV-CAD-), coronary endothelial function was significantly impaired in the HIV+CAD- group, similar to coronary endothelial function in seronegative subjects with established CAD (HIV-CAD+), and associated with high interleukin (IL)-6 levels [38]. These data showed that major coronary endothelial dysfunction is present in PLWH without significant CAD and demonstrated that such endothelial dysfunction can be as severe as in clinical CAD patients.

A study of 266 youths on ART in South Africa showed impaired endothelial function, as assessed via the reactive hyperemic index, compared to seronegative age- and sex-matched controls, and poor endothelial function persisted after 24 months of ART despite viral suppression [39]. There is evidence of increased arterial stiffness and impaired cerebral vasoreactivity in HIV-infected patients associated with increased immune activation and accelerated cellular senescence [40,41,42,43,44], and although patients with high viral loads and/or AIDS were more likely to have higher arterial stiffness, patients on ART also showed increased arterial stiffness [40,42], which suggests that ART use did not improve endothelial function.

### 2.2. Circulating Markers of Endothelial Activation and Vascular Dysfunction in PLWH

HIV infection and endothelial activation can induce the release of endothelial granule contents such as von Willebrand factor (vWF), proinflammatory and prothrombotic factors, and the shedding of cellular adhesion molecules such as intercellular adhesion molecule 1 (ICAM-1), vascular cell adhesion molecule 1 (VCAM-1), and E-selectin [45,46,47]. HIV-infected patients showed persistent high plasma levels of markers of endothelial activation, including vWF [48], soluble (s)VCAM-1, sICAM-1, E-selectin, and fractalkine [49,50,51,52,53,54], as well as markers of monocyte activation (sCD163, sCD14, and CD16+ monocytes), with a direct correlation identified between the levels of endothelial and monocyte activation markers [49]. A study of HIV+ women showed severe endothelial dysfunction associated with increased microvascular oxidative stress, as well as reduced nitric oxide and arginine levels [55]. Compared to seronegative healthy controls, PLWH (both treatment-naïve individuals and those on ART) also showed higher plasma levels of asymmetric dimethylarginine (marker of endothelial dysfunction) associated with viral load, sCD14, D-dimer, and low CD4 counts [56].

Markers of systemic, coronary, and vascular inflammation such as high-sensitivity C-reactive protein (hsCRP); sVCAM-1, sICAM-1, and plasminogen activator inhibitor-1; and soluble tumor necrosis factor receptor-1, sE-selectin, sP-selectin, and sCD40L persist in both naïve HIV-infected patients and PLWH on suppressive ART and are associated with an increased risk of cardiovascular events and coronary heart disease [50,51,52,53,57,58,59]. In a study of 954 HIV+ adults in West Africa, pre- and post-ART, the risk of death was significantly associated with higher baseline plasma sVCAM-1 [52]. A study of 112 HIV+ patients on long-term ART, virologically suppressed, and demographically matched seronegative controls showed that after controlling for cardiovascular disease (CVD) risk factors, HIV+ subjects had higher plasma levels of sCD163 (marker of monocyte activation), sVCAM-1, and sICAM-1 (markers of endothelial activation and injury) [60]. An assessment of endothelial function in PLWH [39,40] also showed that poor endothelial function and increased arterial stiffness was associated with increased plasma sCD14 levels, a marker of monocyte activation [39]; positively correlated with age and platelet counts; and negatively correlated with red blood cell counts [40]. CD4+ and CD8+ cells were similar in patients with and without endothelial dysfunction [40]. In a study of 200 HIV+ patients and seronegative controls, after adjusting for demographics and CVD risk factors, low levels of circulating endothelial progenitor cells (circulating CD34+/KDR+ or CD34+/VE-cadherin+ cells) were associated with ART use and increased cIMT, a measure of sub-clinical atherosclerosis [61]. In a comparative study of HIV+ subjects, both treatment-naïve individuals and those on ART, and seronegative controls, ART use was associated with increased cholesterol, triglycerides, and impaired endothelial function, as well as lower/decreased brachial artery flow-mediated vasodilation (FMD) [62,63,64]. Low FMD of the brachial artery is strongly predictive of future cardiovascular events [65,66].

### 2.3. ARV Drug Classes, Vascular Dysfunction, and Markers of Vascular Diseases in PLWH

Protease inhibitors (PIs) are one the most studied drug classes in relation to endothelial injury, vascular function, and vascular diseases in PLWH. In a study of HIV+ patients on ART and healthy seronegative controls, the HIV+ group had higher cIMT, and patients taking PIs had significantly higher cIMT than patients on non-PI-based ART [37]. Patients on PIs also had significantly higher blood pressure, cholesterol, and triacylglycerols, and regression analyses showed that use of PIs and duration of HIV disease were significant predictors of cIMT [37]. In another study comparing PLWH on PIs and non-PI-based ART, subjects on PIs had significantly higher levels of cholesterol (including low-density and very-low-density lipoproteins), triglycerides, and endothelial dysfunction (impaired FMD), which suggests that the use of PIs was associated with increased levels of atherogenic lipoproteins, and these lipoprotein levels predicted endothelial dysfunction [67].

A study of PLWH showed endothelial impairment with lopinavir/ritonavir (LPV/r) and atazanavir (ATV) regimens. A study of 90 HIV+ children on ART and with undetectable viral loads showed that current or past treatment with LPV/r was associated with increased plasma markers of endothelial activation (angiopoietin-2, soluble vascular endothelial growth factor-1, and soluble endoglin) and systemic inflammation [68] (Table 2). A study of PWLH initiating ATV/r and LPV/r showed that both regimens improved endothelial function (cIMT and FMD) after 24 weeks of ART but worsened endothelial function (cIMT and FMD) at 96 weeks, especially with LPV/r, compared to ATV/r regimens [69]. Indinavir (IDV) impairs endothelial function [70,71]. Some studies showed lower cardiovascular risk with ATV/r compared to darunavir (DRV)/r. A longitudinal study of 119 HIV+ naïve patients starting ATV/r, DRV/r, or efavirenz (EFV)-based regimens showed that over a 12-month follow-up, blood lipids and markers of endothelial dysfunction (sICAM-1 and sVCAM-1) increased for all three regimens, but patients on DRV/r-based regimens had significantly higher cIMT values compared to patients on EFV-based ART, which suggests an increased risk of developing subclinical atherosclerosis with DRV/r [72]. However, EFV-based ART has been shown to reduce FMD and impair endothelial function. A prospective study of PLWH on PI-based regimens and emtricitabine (FTC)/tenofovir (TDF)/EFV showed worse endothelial function (significantly reduced FMD) at both 6 and 12 months in patients on EFV-based ART compared to patients on PI-based ART [73].

Abacavir (ABC) use has also been associated with impaired endothelial function. In a study of 61 PLWH on long-term ART (30 on ABC-based regimens), with a median of 18 years of HIV infection and undetectable viremia, FMD was significantly lower among ABC users compared to patients not taking ABC, and current ABC use was independently associated with impaired endothelial function (lower FMD) [65]. Other studies also showed that the use of ABC was associated with a higher risk of experiencing cardiovascular events [105]. A study comparing patients on ABC-, TDF-, and azidothymidine (AZT)-based regimens showed similar levels of IL-6 and hsCRP (inflammation markers) and D-dimer (coagulopathy marker) among the three groups; FMD was similar for the ABC and TDF groups and higher for the AZT group [106]. A prospective study comparing virologically suppressed patients on ART (FTC/TDF/EFV), treatment-naïve PLWH, and healthy seronegative controls matched for demographics found no difference in FMD between the three groups [112], although naïve patients had significantly higher plasma levels of markers of endothelial activation (sVCAM1) [112,113], monocyte activation (sCD163), and inflammation (soluble tumor necrosis factor receptor-2) [112].

### 2.4. Effects of Changes in ART Regimens on Endothelial Activation and Vascular Function in PLWH

A clinical trial of virologically suppressed HIV+ patients randomized to continue existing PI (non-ATV)-based ART or change to ATV-based regimens and followed up for 24 weeks showed that switching to ATV did not improve endothelial function, even though it significantly improved serum lipids [96]. Another clinical trial of 90 PLWH randomized to ATV and non-ATV regimens showed that after one year of treatment, ATV did not improve arteries’ endothelial function (no changes in either FMD or vasodilation), even though ATV significantly increased plasma antioxidant capacity and reduced hsCRP levels [97].

Other human studies also investigated whether switching to integrase strand transfer inhibitors (INSTIs) improves endothelial function. In a clinical trial of PLWH on different ART regimens and at least one year with undetectable viral load randomized to switch to raltegravir (RAL), 24 weeks of RAL treatment did not change endothelial function, and current use of ABC was associated with endothelial dysfunction (lower FMD) [107]. However, in a second study of patients with suppressed viremia, switching from PIs to RAL significantly reduced FMD (worsen endothelial function) after 8 weeks of RAL treatment compared to the control/no-switch group, even though RAL significantly decreased total cholesterol, low-density lipoprotein, and triglyceride levels [123]. These data showed that switching from PIs to RAL reduced endothelial function while decreasing plasma lipids. A randomized clinical trial switching HIV+ patients receiving TDF/FTC/EFV to TDF/FTC/RAL showed no significant differences in FMD after 24 weeks, suggesting that switching from EFV to RAL did not improve endothelial function, and RAL significantly increased sCD163 levels, a marker of monocyte activation [114]. Other studies showed that the use of INSTIs or switching to INSTI-based regimens also decreased proatherogenic lipids [135,136,137,138] but did not change CVD risks [139,140].

### 2.5. ART Duration, Endothelial Activation, and Vascular Function in PLWH

A study of naïve HIV+ subjects starting ART showed improvement in endothelial function (increased FMD) after 4 to 24 weeks on ART [PI(LPV/r)-based, NRTI-based, and non-nucleoside reverse transcriptase inhibitors (NNRTIs) (EFV)-based regimens], with decreased viremia correlating with higher FMD [74,75]. The short-term (1 month) use of etravirine (ETR) did not change/improve FMD in PLWH [122], and studies of healthy seronegative human treated for 4 weeks with the PIs IDV, ATV, or LPV/r or a placebo showed that IDV impaired endothelium-dependent vasodilation, whereas ATV and LPV/r did not do so [70,76].

PLWH have high plasma sVCAM-1, sICAM-1, vWF, D-dimer, tissue plasminogen activator inhibitor-1, and hsCRP, correlating with a higher viral load [45,75,141,142,143,144,145]. The levels of some of these markers of vascular activation (vWF, sICAM-1, sVCAM-1) decreased after 2 to 24 months on ART [141,146] and increased after ART interruption in correlation with viral rebound and T-cell activation [45]. However, sVCAM-1 levels remained high after the resumption of ART and viral suppression [45]. ART did not decrease other cellular activation markers such as P-selectin [141,144] or tissue plasminogen activator inhibitor-1 [142]. PLWH showed immune activation associated with increased levels of circulating ECs and decreased endothelial progenitor cells; these endothelial changes were partially restored after 24 weeks on DRV-based ART but not with rilpivirine-based ART [103].

Some studies also showed that although ART improved/reduced the levels of markers of endothelial activation [vWF, ICAM-1, VCAM-1] and monocyte activation (sCD14 and sCD163), these markers remained significantly high following ART compared to healthy seronegative controls [143]. It is also possible that decreased in plasma markers of endothelial activation may not always correlate with improved endothelial function. A longitudinal study of naïve PLWH initiating ART (PI/NRTI or PI/NNRTI regimens) showed that after 3, 12, and 24 months of treatment, both regimens significantly decreased plasma sVCAM-1, sICAM-1, and vWF levels but significantly increased cIMT and arterial stiffness; increases in cIMT and arterial stiffness are indicators of subclinical atherosclerosis and have been shown to predict CVD morbidity and mortality [147]. Furthermore, in most of these studies showing improved/decreased markers of endothelium activation following ART use, patients were assessed after a short time (1 to 24 months) on ART [74,75,141,147]. Considering that PLWH are continuously on ART for decades, the effect of such prolonged use of ARV drugs on the vascular endothelium remains to be determined.

## 3. Evidence from Animal Studies

### 3.1. PIs, NRTIs, Endothelial Activation, and Vascular Function

Studies using several animal models, including porcine, rats, and mice, have shown a link between ART use and endothelial dysfunction. In studies exposing porcine coronary artery rings to various PIs [RTV, LPV/r, saquinavir (SQV), IDV, amprenavir (APV), or nelfinavir (NFV)] [77,78,79,80], RTV, APV, and SQV significantly reduced endothelium-dependent vasorelaxation in porcine carotid arteries, and this was associated with the increased production of superoxide anion, nitrotyrosine, oxidative stress, and the decreased expression of endothelial nitric oxide synthase (eNOS) and nitric oxide in porcine coronary artery ECs [77,78,79]. The involvement of oxidative stress was further demonstrated by studies showing that antioxidants reversed the RTV-induced production of superoxide anions and vasomotor dysfunction [79]. In a study of rats treated for one month with PIs and/or NRTIs (AZT, IDV, or AZT+IDV), animals treated with AZT and AZT+IDV showed significant impairments in endothelial function (major decrease in endothelium-dependent vasorelaxation) associated with increased plasma endothelin-1 [81]. These data showed that although IDV alone did not have major effects, it potentiated AZT-induced vascular dysfunction; thus, it is important to investigate the effects of ARV use individually and in combination. AZT and stavudine have also been shown to impair endothelial function [82]. The treatment of rats with ART (LPV/r/lamivudine (3TC)/AZT) promotes neointima formation and impairs the regenerative capacity of the vascular endothelium [81,82,83]. The exposure of mice and rat aortic rings to AZT increased oxidative stress and superoxide levels and impaired endothelial function/endothelium-dependent relaxation [81,82,84].

### 3.2. INSTIs, Endothelial Activation, and Vascular Function

INSTIs have been implicated in endothelial dysfunction. The treatment of C57/BL6 mice with EFV, dolutegravir (DTG), or bictegravir (BIC) decreased the expression of the endothelial tight junction protein occludin and increased blood–brain barrier (BBB) permeability; DTG caused the highest increase in BBB permeability and also increased IL-1β levels [116]. These data suggest an increased risk of brain inflammation with DTG and corroborate human studies showing central nervous system (CNS) adverse events associated with DTG and BIC use [116,124] and a higher rate of DTG discontinuation (compared to other INSTI-based ART) due to CNS adverse events [125].

### 3.3. NNRTIs, Endothelial Activation, and Vascular Function

NNRTI EFV induced endothelial dysfunction in mice and rats. The administration of EFV to ApoE null mice (for 35 days) increased arterial stiffness but did not increase c-IMT [117]. The exposure of rats’ aortic rings to EFV or EFV-based ART induced oxidative stress and impaired endothelial function (acetylcholine-mediated relaxant response); this was associated with increased oxidative stress and increased poly (ADP-ribose) polymerase (PARP) activity, and PARP inhibitors prevented EFV-induced endothelial dysfunction [92]. EFV treatment induced endoplasmic reticulum (ER) stress and increased oxidative stress and vascular permeability in rats [92] and mice [98,118], including in HIV transgenic mice [98].

### 3.4. C-C Chemokine Receptor Type-5 (CCR5) Antagonists, Cellular Activation, and Vascular Function

The CCR5 antagonist maraviroc (MVC) have been shown to be neuroprotective and to protect the brain vascular endothelium in animal studies [129,130,131]. Studies of non-human primates infected with the simian immunodeficient virus [129] and the HIV-1 trans-activator of transcription (Tat) transgenic mice [130] showed that MVC treatment reduced the activation of brain phagocytes [129] and attenuated Tat-induced neuroinflammation [130]. In an HIV-infected humanized mouse model, MVC treatment was neuroprotective, protected the BBB (prevented HIV-induced loss of the brain endothelial tight junction proteins claudin-5, zonula occludens [ZO]-1 and ZO-2), and reduced leukocyte infiltration into the brain [131].

## 4. Evidence from In Vitro Studies

### 4.1. Studies Showing Adverse ARV Effects on Human and Animal ECs

#### 4.1.1. PIs

Several studies have investigated the effects of ARVs on human ECs, including on human brain microvascular ECs [118], human aortic ECs, human umbilical vein ECs (HUVEC) [84,104], human dermal microvascular ECs, human pulmonary arterial ECs, and human coronary artery ECs [119]. These studies showed that the exposure of human ECs to PIs, including SQV, IDV, LPV, RTV, and ATV, significantly decreased eNOS; increased superoxide levels; increased ROS and reactive nitrogen species; induced mitochondrial dysfunction, inflammation, and premature senescence; decreased endothelial tight junction (ZO-1, claudin-1, occludin) and adherent junction (junctional adhesion molecule-1) proteins; and increased endothelial permeability [78,79,84,85,86,87]. The exposure of human pulmonary artery ECs to LPV/r decreased eNOS and nitric oxide; increased superoxide, ROS, inflammation, and cellular senescence; and increased sICAM, sVCAM, and endothelin-1 [79,88,89]. Another study showed that the exposure of human brain ECs to physiological levels of SQV, IDV, NFV, and RTV did not alter cell viability but significantly increased ROS and matrix metalloprotein-2 (MMP-2) levels [93].

#### 4.1.2. NNRTIs

NNRTIs, alone or in combination with PIs, also affect ECs. EFV-induced endothelial dysfunction and mitochondrial toxicity increased superoxide and vascular permeability, and this was associated with the disruption/downregulation of endothelial tight junction and adherent junction proteins [99,119] and increased infiltration of leukocytes into the CNS [120]. EFV also increased CD11b, CD11c, and CD18 in monocytes and neutrophils, resulting in increased leukocyte–endothelial adhesion in vitro and in vivo [121]. The exposure of rats’ ECs [92], human coronary artery ECs [119], human brain microvascular ECs [118], and HUVEC [104] to EFV increased oxidative stress and markers of ER stress and autophagy, induced apoptosis and necrosis [92], decreased endothelial tight junction (ZO-1, claudin-1, occludin) and adherent junction (junctional adhesion molecule-1) proteins, and increased endothelial permeability [118,119]. The combination of EFV with NFV further increased oxidative and ER stress in HUVEC [104]. The exposure of human coronary artery ECs and human aortic ECs to RTV [94], IDV, or NFV [99], alone or in combination with EFV, significantly decreased eNOS mRNA and proteins [94], increased cellular oxidative stress and PARP activity [87], induced ROS, and dose-dependently increased leukocyte adhesion to ECs [99].

#### 4.1.3. NRTIs

NRTIs were associated with increased endothelial stress and injury. The exposure of HUVEC [84], human pulmonary arterial ECs, and porcine pulmonary arteries [88] to ABC, 3TC, and AZT (with and without IDV) decreased eNOS, increased oxidative stress and superoxide and mitochondria dysfunction (increased mitochondrial-specific superoxide and decreased mitochondrial membrane potential), and increased lactate release and cell death [84,88]. The exposure of human aortic ECs, coronary artery ECs, and human brain microvascular ECs [100,101] to AZT (with and without IDV) disrupted endothelial tight junction and gap junction proteins and decreased trans-endothelial electric resistance [100]. These studies also showed that AZT’s effects on the endothelium were similar to HIV-1- and glycoprotein 120 (gp120)-induced effects [100]. AZT also induced oxidative stress (significantly increased oxidized glutathione redox potential and total cellular superoxide) and mitochondrial dysfunction (increased mitochondrial-specific superoxide and decreased mitochondrial membrane potential) and increased lactate release and cell death [101]. Other studies also showed that AZT induced mitochondrial dysfunction in ECs, significantly reduced mitochondrial respiration, decreased adenosine triphosphate production, and impaired mitochondrial membrane potential in cultured ECs [84]. The exposure of ECs to AZT increased endothelin-1 [110] and induced the formation of intracellular gaps [100] and endothelial dysfunction, and this was associated with increased superoxide levels [81,82,84]. The exposure of a brain endothelial cell line (hCMEC/D3 cells) to AZT or IDV also dose-dependently increased ROS, altered glutathione, and induced mitochondrial dysfunction (loss of mitochondrial membrane potential). The combination of AZT and IDV further increased apoptotic markers (increased the release of cytochrome C and procaspase-3), and these effects were reversed by the antioxidant N-acetyl cysteine [102].

The exposure of HUVEC to TDF and FTC induced premature senescence associated with increased inflammation, oxidative stress, altered eNOS, and endothelial activation, and the exposure of healthy astrocytes to senescent HUVEC induced senescence in astrocytes, suggesting a paracrine effect of endothelial senescence on other cell types [115]. Such ARV-induced senescence of CNS cells (either directly or indirectly) could contribute to premature cellular aging, neurodegeneration, and cognitive impairment. In fact, there is evidence of a link between the premature senescence of CNS cells and the development/presence of neurodegenerative diseases such as Alzheimer’s disease and Parkinson’s disease [148,149,150]. The exposure of ECs to gp120, inflammatory cytokines such as tumor necrosis factor-α, and oxidative stress have also been shown to cause premature senescence [151,152,153].

#### 4.1.4. INSTIs

INSTIs also affect vascular ECs. The exposure of hCMEC/D3 and primary mouse ECs to EFV, DTG, or BIC decreased endothelial tight junction proteins (claudin-5 and occludin) and increased nitric oxide synthase-2 and proinflammatory cytokines (IL-6, IL-8, and IL-1β), with a major increase in inflammation observed in cells exposed to DTG [116].

### 4.2. Studies Showing No Adverse Effects or Protective Effects of ARVs on ECs

#### 4.2.1. PIs, NRTIs, and NNRTIs

The exposure of HUVEC, human aortic ECs, and human dermal microvascular ECs to the PIs LPV, ATV, or RTV induced heme-oxygenase-1 [90], an inducible enzyme that break down heme and has antioxidant and anti-inflammatory properties [154,155]. Whereas the PI RTV and NNRTI EFV reduced eNOS and increased oxidative stress in human coronary artery ECs [92], the exposure of human coronary artery ECs, HUVEC, and rat ECs and aortic rings to the NRTIs TDF, FTC, or ABC did not increase oxidative stress or proinflammatory genes [87,108]; alter VCAM1, ICAM1, MCP1, RANTES, or IL-6 levels [109]; or impair endothelial function [92].

#### 4.2.2. CCR5 Antagonists

CCR5 antagonists, including MVC and TAK-779, have been shown to be protective of vascular ECs. Primary human brain microvascular ECs express CCR5, and CCR5-neutralizing antibodies blocked gp120-induced increases in BBB permeability and monocyte migration and prevented the gp120-induced release of intracellular calcium [132]. Studies also showed that CCR5 was partially involved in HIV-1 binding to human brain microvascular ECs, and CCR5 neutralizing antibodies diminished HIV-induced monocyte adhesion and transendothelial migration [133]. There is also evidence that CCR5 and the cytoskeleton are involved in endothelial–mononuclear phagocyte interactions, adhesion, and HIV-1 infection. HIV-1 activates cytoskeletal proteins during monocyte–endothelial interactions, and the CCR5 antagonists MVC and TAK-799 prevented the HIV-induced upregulation and phosphorylation of cytoskeleton-associated proteins, prevented HIV-1 infection of macrophages, and diminished the viral-induced adhesion of monocytes to human brain microvascular ECs [13].

Several other studies also showed beneficial effects of MVC in PLWH and human ECs. MVC decreased inflammation in human coronary artery ECs [134]; significantly reduced IL-6, sICAM1, and sVCAM-1 in PI-treated PLWH [126]; and reduced arterial stiffness and inhibited neointimal development in human coronary artery [127]. MVC improved endothelial function (significantly improved brachial FMD, cIMT, and carotid–femoral pulse wave velocity) in PLWH [126,128] and decreased circulating endothelial microparticles (a marker of endothelial activation) [128]. MVC also inhibited any ATV/r-induced increase in leukocyte-endothelial adhesion; prevented/reduced ATV/r-induced increases in IL-6, IL-8, sICAM1, and sVCAM1 secretion; and abrogated the ATV/r-induced senescence of human coronary ECs [87].

## 5. Potential Mechanisms of ARV-Induced Endothelial Activation and Vascular Dysfunction

### 5.1. PIs

PI-induced endothelial injury often involves increased oxidative stress and the production of ROS and reactive nitrogen species, and antioxidants such as vitamin-E and N-acetyl cysteine have been shown to diminish PI-induced endothelial injury [93,102]. The PI-induced dysfunction of porcine coronary artery ECs is associated with activation of mitogen-activated protein kinases [78,79]. NFV induced ROS and MMP-2 in human brain ECs via the nuclear factor kappaB (NFκB) and Notch4 pathways, and this was associated with increased Notch4 translocation to nuclear intracellular domains. The involvement of oxidative stress was further demonstrated by studies showing that vitamin-E prevented PI-induced ROS, NFV-induced Notch4 translocation to nuclear intracellular domains, and NFV-induced NFκB activation and MMP-2 expression [93]. ATV/r have also been shown to increase IL-6, IL-8, sICAM-1, and sVCAM-1 secretion human coronary ECs by altering NFκB activation [87]. The exposure of vascular ECs to LPV/r (or Tat) induced endothelial senescence, which was associated with increased microRNA-34a, and the inhibition of this microRNA prevented LPV/r (and Tat)-induced endothelial senescence [91]. The exposure of human dermal microvascular ECs to RTV decreased tight junction (ZO-1, occludin, claudin-1) mRNA by up to 60% and significantly increased endothelial permeability via mechanisms involving increased superoxide and the activation of ERK1/2, and treatment with antioxidants or ERK1/2 inhibitors prevented any RTV-induced decrease in tight junction mRNA and proteins, superoxide production, and ERK1/2 activation [86]. The exposure of human or porcine pulmonary ECs to RTV also increased superoxide levels and decreased eNOS and endothelium-dependent vasorelaxation, and antioxidants prevented these RTV-induced effects [95].

### 5.2. NNRTIs

EFV induced ROS, apoptosis, and necrosis; decreased tight junction proteins; and increased the permeability of rat ECs, HUVEC, and human brain microvascular ECs via mechanisms involving the activation of c-Jun N-terminal kinases and NFκB, the induction of heat-shock proteins, and increased ER stress and reduced autophagy [92,98,104,118,119]. EFV-induced impairment of the vascular endothelium involved increased PARP activity and could be prevented via PARP inhibition [92]. In vitro and in vivo studies showed that EFV used alone or in combination with IDV, 3TC, TDF, or FTC induced ER stress and endothelial dysfunction in human brain microvascular ECs and HIV transgenic mice, and this was associated with (1) the upregulation and activation of two ER kinases (protein kinase-like ER kinase and inositol-requiring transmembrane kinase 1-alpha and (2) decreased/dysregulated autophagy (EFV blocked the activity of Beclin-1/autophagy-related gene-14/phosphatidylinositol-3-kinase class-3 complex, a complex linked to phosphatidylinositol-3-phosphate synthesis and the formation of autophagosomes) [98].

### 5.3. NRTIs

It has been demonstrated that TDF and FTC induced the premature senescence of HUVEC, and senescent HUVEC further induced senescence in astrocytes [115]; this suggests a paracrine mechanism of ARV-induced endothelial senescence on other cell types. AZT- and RTV-induced impairment of pulmonary arteries and human pulmonary artery EC function are also mediated by increased oxidative stress, eNOS downregulation and ERK1/2 activation, and the inhibition of ERK1/2 partially blocked RTV- and AZT-induced eNOS downregulation and vasomotor dysfunction [88]. AZT, TDF, and 3TC inhibit angiogenesis and lymphangiogenesis in vitro and in vivo via oxidative stress involving impaired receptor tyrosine kinase (RTK)-mediated endocytosis and maturation [111].

### 5.4. CCR5 Antagonists

The CCR5 antagonist MVC induced protective effects on the brain endothelium via crosstalk with the signal transducer and activator of transcription 1 (STAT1), 3-phosphoinositide-dependent kinase 1 (PDK1), and protein kinase-B (AKT) pathways [16,133]. It has been shown that CCR5 neutralizing antibodies decreased leukocyte adhesion to brain ECs and transendothelial migration by inhibiting the HIV-induced activation of STAT1 and interferon-stimulated response element/interferon-γ-activated site promoter, and this involved crosstalk with PDK1 and AKT [133]. MVC also prevented HIV-induced BBB dysfunction by blocking the activation of cytoskeletal proteins during leukocyte–endothelial interaction [13].

## 6. Conclusions

In summary, current evidence shows that while some ARVs such as the CCR5 antagonist MVC have protective effects on the vascular endothelium, several other ARVs, including PIs, NRTIs and NNRTIs, induce the injury and dysfunction of the vascular endothelium, and this is often associated with the increased release of markers of endothelial activation (from endothelial granules), the shedding of endothelial adhesion molecules, oxidative stress, dysregulated autophagy, inflammation, and cellular senescence. Evidence that ARVs induced senescence in ECs, as well as that senescent ECs further induced astrocytes senescence, shows the paracrine effect of ARV-induced endothelial senescence on other cell types. As HIV virions, viral proteins, inflammatory cytokines, and oxidative stress also cause premature EC senescence, it would be important to determine whether, in the presence of HIV/viral proteins, ARVs protect or potentiate HIV-induced cellular senescence, as well as whether the effects differ based on specific ARVs and/or drug classes.

The vascular endothelium has traditionally been known for its role in angiogenesis, the regulation of vascular permeability, and homeostasis. There is now evidence that in response to stimulation and injury, ECs produce angiocrine factors, and evidence that ECs’ angiocrine function regulates the vascular microenvironment, including leukocyte antigen recognition and immune surveillance, metastasis, cell proliferation, and tissue regeneration. Several comorbid conditions that are prevalent in PLWH, including malignancies, CVD, premature aging, CNS, and metabolic diseases, involve EC activation and injury. It would be important to investigate whether these comorbid conditions involve angiocrine signaling, the potential EC angiocrine signals involved, the surrounding parenchymal or effector cells involved, and whether ARVs, HIV, or viral proteins dysregulate ECs’ angiocrine function. Such studies and the identification of vascular niches involved in angiocrine signaling could help design therapeutics against vascular-mediated comorbid diseases in PLWH.

## 7. Limitations

Limitations of current human studies evaluating ARVs’ effects on the vascular endothelium include treatment duration and timelines. For several studies showing improvement, i.e., decreased markers of endothelium activation following ART use, patients were assessed after a short time (1 to 24 months) on ART. Considering that PLWH are continuously on ART for decades, the effects of such prolonged use of ARV drugs on the vascular endothelium remains to be determined. It also remains to be seen whether ARV drugs that decrease markers of endothelial activation would result in decreased risk of vascular diseases, as some studies showed that the use of INSTIs or switching to INSTI-based regimens decreased proatherogenic lipids but did not change CVD risks. Animal studies showing that IDV alone had no major effect on endothelial function but potentiated AZT-induced vascular dysfunction show that it is important to investigate whether ARV drugs used in combination additively or synergistically affect the vascular endothelium. Newly developed ARV drugs should also be investigated for potential vascular adverse events and association with comorbidities.

## Figures and Tables

**Table 1:** Abbreviations list.

HIV	Human immunodeficiency virus
AIDS	Acquired immunodeficiency syndrome
PLWH	People living with HIV
EC	Endothelial cells
HUVEC	Human umbilical vein endothelial cells
BBB	Blood-brain barrier
CNS	Central nervous system
ART	Antiretroviral therapy
ARVs	Antiretrovirals
ROS	Reactive oxygen species
cIMT	Carotid intima media thickness
CAD	Coronary artery disease
IL	interleukin
vWF	von Willebrand factor
ICAM-1	Intercellular adhesion molecule 1
sICAM-1	Soluble intercellular adhesion molecule 1
VCAM-1	Vascular cell adhesion molecule 1
sVCAM-1	Soluble vascular cell adhesion molecule 1
hsCRP	High-sensitivity C-reactive protein
CVD	cardiovascular diseases
FMD	Flow-mediated vasodilation
NRTIs	Nucleoside reverse transcriptase inhibitors
NNRTIs	Non-nucleoside reverse transcriptase inhibitors
Pis	Protease inhibitors
LPV	lopinavir
RTV (r)	Ritonavir
ATV	atazanavir
IDV	Indinavir
DRV	Darunavir
EFV	Efavirenz
FTC	Emtricitabine
TDF	Tenofovir disoproxil fumarate
ABC	Abacavir
AZT	Abacavir
INSTIs	Integrase strand transfer inhibitors
RAL	Raltegravir
DTG	Dolutegravir
BIC	Bictegravir
ETR	Etravirine
SQV	Saquinavir
APV	Amprenavir
NFV	Nelfinavir
eNOS	Endothelial nitric oxide synthase
3TC	Lamivudine
PARP	poly (ADP-ribose) polymerase
CCR5	C-C chemokine receptor type 5
MVC	Maraviroc
Tat	Trans-Activator of Transcription
Gp120	Glycoprotein 120
MMP-2	Matrix metalloprotein-2
ER	Endoplasmic reticulum
NFkB	Nuclear factor kappaB
STAT-1	signal transducer and activator of transcription 1
AKT	Protein kinase-B
PDK1	3-phosphoinositide-dependent kinase 1

**Table 2:** Antiretroviral drugs investigated for their effects on the endothelium and vascular function.

Antiretrovirals	Human Studies Refs.	Animal Studies Refs.	In Vitro Studies Refs.
**Protease inhibitors**			
Lopinavir	[68,69,70,74,75,76]	[77,78,79,80,81,82,83]	[78,79,84,85,86,87,88,89,90,91]
Ritonavir	[68,69,70,72,74,75,76]	[77,78,79,80,81,82,83]	[78,79,84,85,86,87,88,89,90,91,92,93,94,95]
Atazanavir	[69,70,71,72,76,96,97]		[78,79,84,85,86,87,90]
Indinavir	[70,76]	[77,78,79,80,81]	[78,79,84,85,86,87,88,93,98,99,100,101,102]
Darunavir	[72,103]		
Saquinavir		[77,78,79,80]	[78,79,84,85,86,87,93]
Amprenavir		[77,78,79,80]	
Nelfinavir		[77,78,79,80]	[87,93,99,104]
**NRTIs**			
Abacavir	[65,105,106,107]		[84,87,88,92,94,108,109]
Azidothymidine	[106]	[81,82,83,84]	[81,82,83,84,88,100,101,102,110,111]
Emtricitabine	[73,112,113,114]		[87,92,94,98,108,109,115]
Lamivudine		[81,82,83]	[84,88,98,111]
Tenofovir	[73,106,112,113,114]		[87,92,94,98,108,109,111,115]
Stavudine		[82]	
**NNRTIs**			
Efavirenz	[72,73,74,75,112,113,114]	[92,98,116,117,118]	[87,92,94,98,99,104,116,118,119,120,121]
Etravirine	[122]		
Rilpivirine	[103]		
**Integrase inhibitors**			
Raltegravir	[107,114,123]		
Dolutegravir	[116,124,125]	[116]	[116]
Bictegravir	[116,124]	[116]	[116]
**CCR5 inhibtors**			
Maraviroc	[126,127,128]	[129,130,131]	[13,16,87,132,133,134]

Abbreviations: Refs: references; NRTIs: nucleoside reverse transcriptase inhibitors; NNRTIs: non-nucleoside reverse transcriptase inhibitors.
